# Collagenous colitis and atezolizumab therapy: an atypical case

**DOI:** 10.1007/s12328-020-01276-4

**Published:** 2020-11-05

**Authors:** Antonella Gallo, Rosa Talerico, Luca Novello, Maria Cristina Giustiniani, Ettore D’Argento, Emilio Bria, Massimo Montalto

**Affiliations:** 1 Internal Medicine, UOC Clinica Medica, Fondazione Policlinico Universitario “A.Gemelli” IRCCS, Largo Gemelli, 8, 00168 Rome, Italy; 2 Anatomia Patologica, Fondazione Policlinico Universitario “A. Gemelli” IRCCS, Rome, Italy; 3 Comprehensive Cancer Center, Fondazione Policlinico Universitario “A. Gemelli” IRCCS, Rome, Italy; 4 Università Cattolica del Sacro Cuore, Rome, Italy

**Keywords:** Immunotherapy, Diarrhea, Cancer, Microscopic colitis

## Abstract

Immune checkpoint inhibitors such as anti-CTLA-4 (cytotoxic T-lymphocyte-associated protein 4), anti-PD-1 (programmed cell death protein 1), and PD-L1 (programmed cell death protein-ligand 1) are emerging drugs that have radically changed treatment and prognosis of different types of tumors. However, despite their considerable benefits, immune checkpoint inhibitors are associated with numerous side effects involving several organs. Gastrointestinal toxicities represent some of these most common adverse events. While clinical presentation usually ranges from mild diarrhea to life-threatening colitis, typical endoscopic and histologic findings of immune-mediated colitis often resemble those of inflammatory bowel diseases. However, less common patterns are lymphocytic colitis and, rarely, collagenous colitis. Physician and pathologists must be aware of the wide spectrum of clinical and histological findings that may be encountered in immune-related gastro-intestinal toxicities. We report a rare and atypical case of collagenous colitis occurred in a woman affected by stage IV lung adenocarcinoma, on atezolizumab therapy.

## Introduction

Immune checkpoint inhibitors (ICPIs) such as anti-CTLA-4 (cytotoxic T-lymphocyte–associated protein 4), anti-PD-1 (programmed cell death protein 1), and PD-L1 (programmed cell death protein–ligand 1) are emerging drugs that have radically changed treatment and prognosis of different types of tumors^1^. However, despite their considerable benefits, ICPIs are associated with numerous side effects involving several organs; gastro-intestinal toxicity is one of them [1]. Typical endoscopic and histologic findings of immune-mediated colitis often resemble those of inflammatory bowel diseases (IBDs), but less common patterns have been described. Physicians and pathologists must be aware of the wide spectrum of clinical and histological findings that may be encountered in immune-related gastro-intestinal toxicities.

We report a rare and atypical case of collagenous colitis occurred in a woman affected by stage IV lung adenocarcinoma, on atezolizumab therapy.

## Case report

A 57-year-old woman with IV stage pulmonary adenocarcinoma (G3 pT1b pN0 M + , PD-L1 40%, EGFR/ALK WT, ROS1-) was admitted to the Emergency Department (ED) of our Hospital and then hospitalized to the Department of Internal Medicine for diarrhea (up to 15 episodes of watery stools per day), asthenia, fatigue, loss of appetite, without fever, or other associated symptoms.

**Fig. 1:**
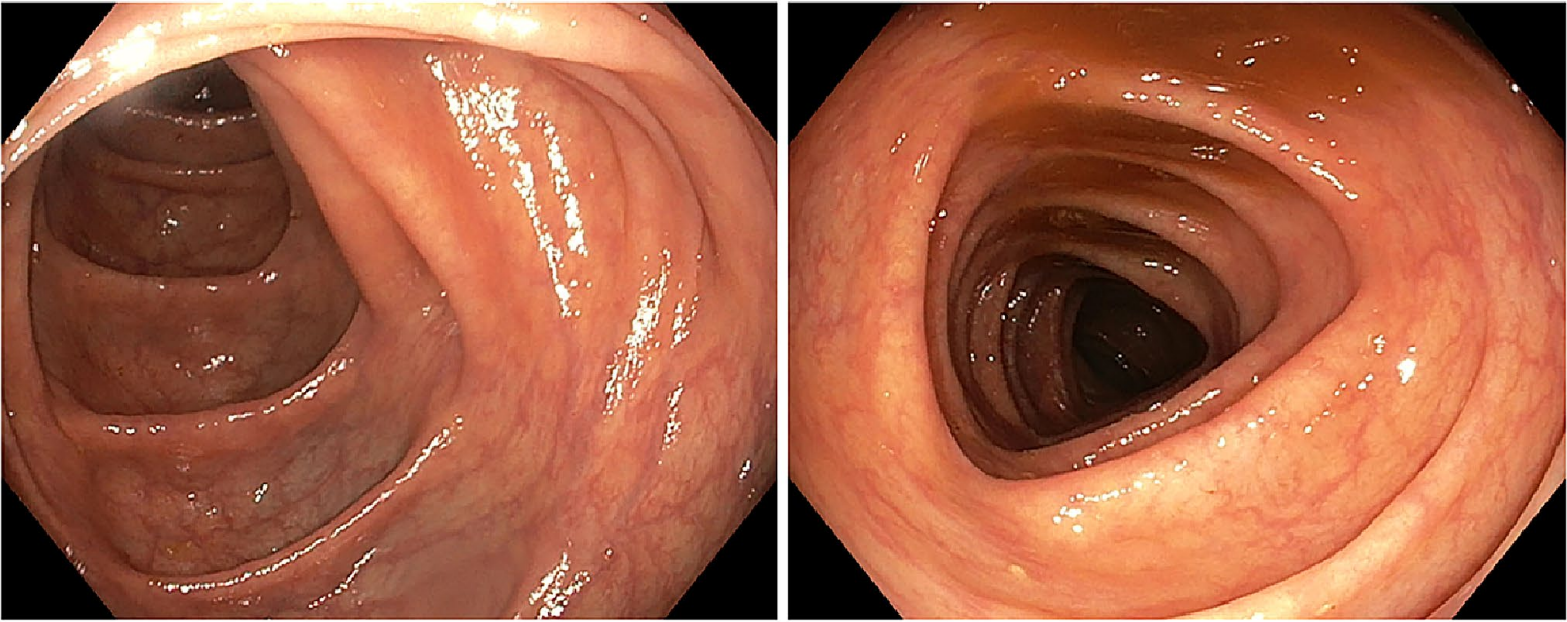
Endoscopic features of our immune checkpoint inhibitor-induced colitis showing a colic mucosa

**Fig. 2:**
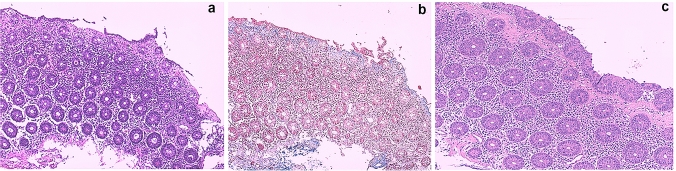
Histology features of our immune checkpoint inhibitor-induced colitis. **a** Right colic dx mucosa with detachment of surface epithelial cells, thickened amorphous hyaline eosinophilic subepithelial band (thickness 24 µm), and active inflammation with intraepithelial neutrophilic granulocytes. The same features were found in left colon biopsy (hematoxylin–eosin 10 ×). **b** The abnormal collagen band is confirmed by Masson’s trichrome stain. **c** Follow-up colonoscopy with random biopsy showing persistence features of collagenous colitis with thickened amorphous hyaline eosinophilic subepithelial band, without signs of acute inflammation (hematoxylin–eosin 10 ×)

Past medical history dates back to 2015 when the patient underwent medial lobe lobectomy plus hilar lymphadenectomy for tumor resection. In 2016, a left parieto-occipital craniotomy was performed for resection of an occipital cerebral metastasis, followed by radio-chemotherapy with carboplatinum and paclitaxel. In December 2018, she started atezolizumab therapy due to primary neoplasia relapse. Two weeks later, the patient presented not bloody diarrhea (no more than five episodes of watery stools/day). She did not experience any fever, chills, vomiting, abdominal pain, and cramps. Loperamide, probiotics, and rifaximin were administered without any benefit. Since worsening of diarrhea, up to 15 watery stools per day, she was hospitalized in February 2019. Due to suspicion of severe immune-related gastro-intestinal toxicity, atezolizumab was discontinued. At that time, the patient was not on chronic therapy, in particular not with proton pump inhibitor (PPIs), histamine type 2 receptor antagonists (H_2_ blockers) neither with non-steroidal antiinflammatory drugs (NSAIDs) or serotoninergic agents, all of them usually being considered as potential triggers of development of microscopic colitis in susceptible individuals [2]. On physical examination, the patient appeared fatigued, but vital signs were normal. Blood chemistry showed no remarkable findings, except only for a slight increase of serum creatinine and low levels of serum potassium. Stool exams were negative for bacterial and parasite infection. Candida was found and treated with oral nystatin without clinical benefit; therefore, an infective cause of diarrhea was recognized as less probable. Gastroscopy and colonoscopy (Fig. 1) did not show significant macroscopic findings. However, since the high clinical suspicion of colitis, endoscopist made random colon biopsies after consult with Oncologist and Clinical Physicians. Microscopic examination of colonic mucosa showed expansion of lamina propria by lymphoplasmacytic infiltrate, intraepithelial neutrophils, crypt abscesses, and a thickened subepithelial collagen layer enhanced, thus resembling a form of collagenous colitis mixed to elements typical of acute neutrophilic inflammation (Fig. 2a–b). Methylprednisolone (1 mg/kg/day) treatment was started, and few days later, diarrhea decreased significantly (up to two episodes per day).

Steroid therapy was slowly tapered within 6 months, but the attempt to completely discontinue the treatment led to reappearance of intestinal symptoms. After 1 year, patient is well on methylprednisolone 4 mg/day and neoplastic picture is unchanged, despite discontinuation of oncologic therapy. However, at follow-up colonoscopy, histological examination still shows a collagenous colitis picture, though without signs of acute neutrophilic inflammation (Fig. 2c).

**Fig. 3:**
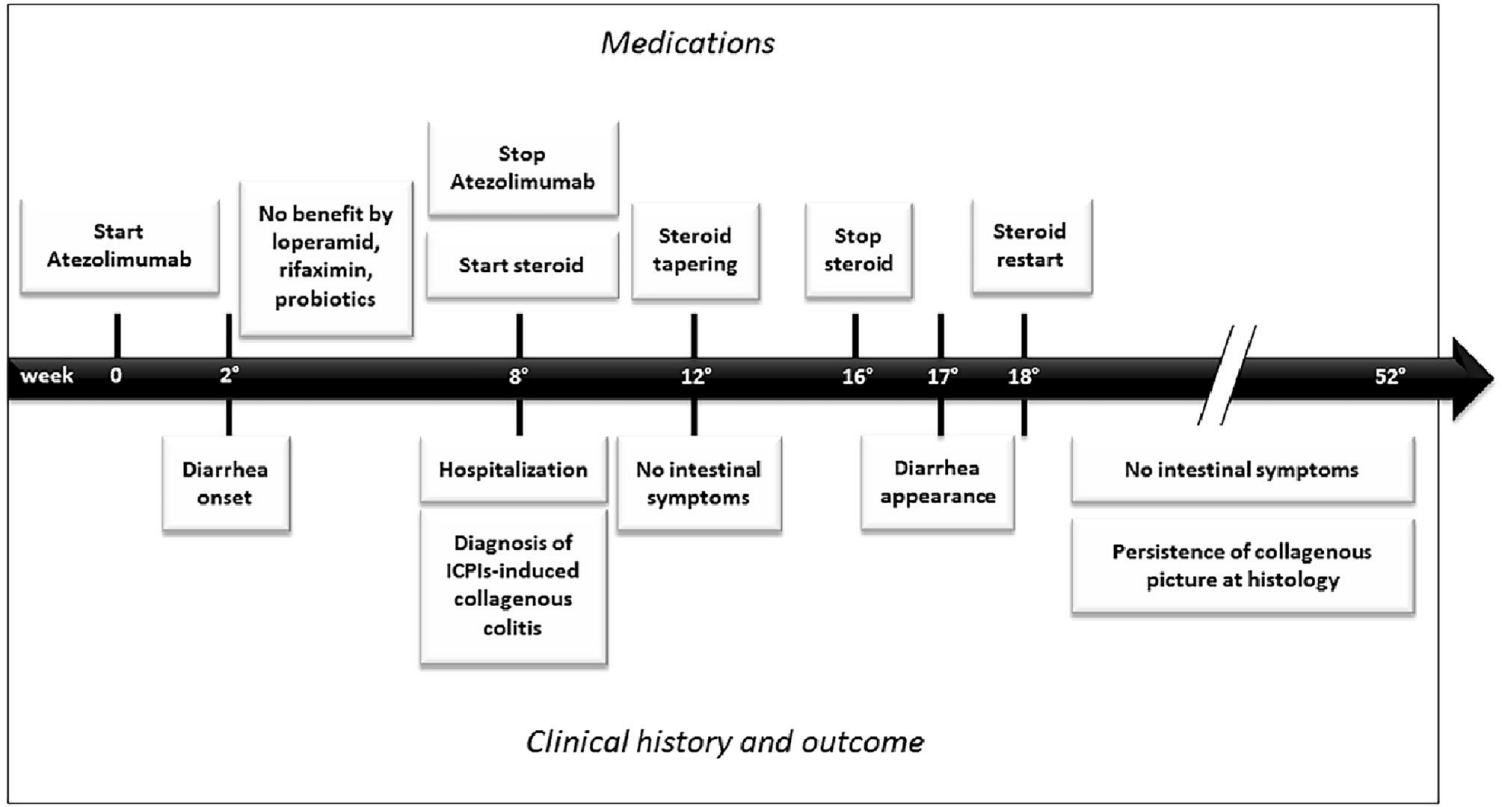
A timeline showing clinical course of disease in our patient

Clinical course of disease in our patient was summarized in a timeline (Fig. 3).

## Discussion

Gastrointestinal toxicity is among the most severe and common immune-related adverse event (irAE) related to the increased use of ICPIs^3^. According to the National Cancer Institute's Common Terminology Criteria, irAEs of the lower gastro-intestinal tract may present as mild-to-moderate transient diarrhea (grade 1) to life-threatening consequences with urgent intervention indicated (grade 4) or death (grade 5)^1^. ICPI-induced diarrhea occurs in up to 30% of patients in clinical trials [3].

In our report, the patient only complained of diarrhea, first classified as grade 2. However, when stool frequency worsened, colonoscopy became necessary to confirm the suspicion of immune-induced colitis, even though the absence of abdominal pain, rectal bleeding, or the presence of mucus in stools. No evidence of macroscopic findings at endoscopy was found. It was only by random biopsies, performed on an apparently healthy mucosa, that intestinal inflammation became manifest.

Currently, mechanisms underlying development of gut inflammation and colitis in a subgroup of patients on ICPIs therapy still remain quite unclear [3]. However, it has been widely shown that gut bacteria represent fundamental mediators of ICPIs toxicity by dysregulation of gastro-intestinal mucosal immunity and persistent T-cell activation, thus resulting in a pro-inflammatory state and occurrence of autoimmune-type manifestations [1].

Histological pattern of immune-mediated colitis is usually similar to other types of colitis, mainly active IBDs [4]; however, other less common patterns including lymphocytic and, very rarely, collagenous colitis have been described [4].

It has been widely suggested that microscopic colitis, both in its collagenous and lymphocytic form, recognize autoimmune mechanism for its development, although real pathophysiology is not completely understood ^2^. Based on these similarities on immune dysregulation, a link between ICPI-intestinal toxicity and microscopic colitis has been recently supposed, mainly in subjects with a genetic predisposition [5–7].

Chen et al. reported three cases of lymphocytic colitis-like pattern among eight patients developing colitis while on anti PD-1 monotherapy [5]. An anti-PD-1-associated collagenous colitis has been reported in a melanoma patient on pembrolizumab [6]. In a recent retrospective review of colonic biopsies of 31 patients with suspected gastro-intestinal irAEs [7], two cases of collagenous colitis pattern were reported, without signs of neutrophilic activity.

Usually, collagenous and lymphocytic colitis are histologically characterized by normal crypt architecture, increased mononuclear inflammation in lamina propria, absence of neutrophils, and increased intraepithelial lymphocytes [8]. Presence of microscopic typical features of active IBDs, like neutrophilic cryptitis and crypt abscesses, have been also reported, although rare [8]. In our case, typical features of a collagenous colitis coexisted to sign of acute neutrophilic inflammation, which is a very atypical microscopic pattern of immune-mediated colitis.

As regarding endoscopic picture, microscopic colitis is typically characterized by normal findings. However, mucosal macroscopic abnormalities have been reported, such as erythema and edema. Recently, Choi et al. [9] conducted a retrospective review among 65 patients with microscopic colitis, showing that the subgroup of patients on ICPI therapy showed more frequently endoscopic abnormalities (exudates, granularity, erythema, loss of vascularity, and erosions/ulcerations), with respect to patients with a non-ICPI-induced microscopic colitis. According to the authors, this may be expression of a higher level of inflammation, and consequently, of a more aggressive disease, also confirmed by requirement of a more potent immunosuppressive regimen for effective disease control [9].

Our patient did not show macroscopic finding at endoscopy; however, this report just underlines the importance to perform biopsy when a case of immune-related colitis is suspected. In fact, despite the most of patients with active inflammation had also high-risk features at endoscopy [10, 11]; however, a negative macroscopic pattern did not exclude the possibility of a consistent inflammatory microscopic picture [10]. Moreover, accurate clinical history and differential diagnosis are fundamental to confirm hypothesis of an irAE. As in our patient, exclusion of other risk factors, associated with a compatible time correlation between ICPIs exposure history and the aforementioned clinical and histological pictures, supports suspicion in diagnosing an immune-mediated from other forms of colitis.

Therefore, a multidisciplinary approach among gastroenterologists, oncologists, and pathologists is necessary to early recognize and manage these patients, since the fewer days elapse from the onset of symptoms and starting of treatment, the more likely a significant clinical improvement can occur [9].

Our patient was treated with systemic corticosteroids and checkpoint inhibitor therapy was discontinued, as suggested by current guidelines [12]. Despite the resolution of gastro-intestinal symptoms, however, this represents a typical case where many concerns still rise about the following strategies to adopt, as regarding the choice to permanently or transiently discontinue the checkpoint inhibitor therapy, the need to continue steroids, and the best follow-up to suggest. In fact, repeat endoscopy of our patients still showed persistence of a collagenous colitis picture, but no more signs of acute inflammation. Up to now, universal guidelines of long-term evaluation are still lacking.

At this regards, Wang et al. [11] showed that repeat endoscopy of 13 patients revealed persistent endoscopic and histological inflammation in most of them, independently from clinical recurrence of diarrhea. In particular, it is likely that long-term follow-up may reveal transition from an acute to a chronic or different inflammatory pattern, suggesting that ICPI therapy has a long-term effect on the immune system. However, this hypothesis only derived from very few cases and more clinical data are needed to better understand the behavior of immune-induced colitis and, consequently, to give practical guidelines on long-term management of checkpoint inhibitor therapy.
